# Differences in Skin Health Outcomes according to Physical Activity Level among Korean Female College Students

**Published:** 2019-10

**Authors:** Jeong-Hyun LEE, Yeona KIM, Wi-Young SO

**Affiliations:** 1. Department of Beauty Art, Youngsan University, Busan, Republic of Korea; 2. Sports and Health Care Major, Korea National University of Transportation, Chungju-si, Republic of Korea

## Dear Editor-in-Chief

The primary roles of sweat are regulation of body temperature, electrolyte balance through lactic and amino acid transfer, and providing the skin with a natural moisturizing effect (1). In addition, antimicrobial peptides and immune globulin that are contained in sweat complement the skin's function as the first line of defense between our body and environment (2). Increasing sweat production in the skin (e.g., the face) by increasing physical activity can help the skin to expel wastes through the pores.

Exercise had a positive effect on various skin health outcomes (3). However, there are very few studies on the relationship between physical activity level (PAL) and skin health outcomes in Republic of Korea.

Therefore, this study aimed to examine the relationship between PAL and skin health outcomes in Korean female college students.

This study included 120 Korean female college students with no history of drug intake, drinking, or smoking. The study was conducted in July 2019 in a beauty & healthcare center at Youngsan University, Busan, Republic of Korea.

All study procedures were approved by the Institutional Review Board of Youngsan University, Busan, Republic of Korea (YSUIRB-201907-HR-054-02) and the participants provided informed consent for participation in the study.

The PAL of each participant was determined using the International Physical Activity Questionnaire (4). To measure skin health outcomes, a JANUS-2 system (Janus imaging system, PSI Co. Ltd, Seoul, Republic of Korea) skin analyzer was used. The JANUS skin analyzer fixes the face in a specific location, blocks external sources of light, takes a picture of the face in three separate conditions (natural light, polarized light, and ultraviolet light) using a 10 megapixel Digital Single-Lens Reflex Camera, saves the picture as a digital image, and analyzes the stored image using image processing. Pores, wrinkles, pigmentation, pigmentation with ultraviolet (UV) light, pigmentation with polarizing light, sebum level, porphyrin content, and skin tone were measured in this study.

Each participant's face was washed using water, all makeup was removed, and the face was allowed to dry naturally before measuring the skin condition to minimize errors. The participant's forehead and chin were fixed to the equipment after the hair was pulled back from the face with a headband 15 minutes after drying.

All results are presented as mean ± standard deviation. According to the central limit theorem, if the number of subjects is over 30, the study will be approximately normally distributed and will have reliability (5). Therefore, we divided the participants into three groups of 40 each. Data analysis was performed using one-way analysis of variance, and the post-hoc test was conducted using Tukey test. Statistical significance was set at p*<*0.05, and all analyses were performed using PASW Statistics 18.0 (IBM Corp., Armonk, NY, USA).

The participant characteristics are shown in Table 1. The differences among 3 PAL groups according to skin health outcomes are shown in Table 2. The results showed significant differences in only wrinkles (*P*=0.004); there were no significant differences in pores, pigmentation with UV light, pigmentation with polarizing light, sebum level, porphyrin content, and skin tone among the 3 PAL groups.

**Table 1:** Participant characteristics

***Variables***	***Low PAL group (n=40)***	***Middle PAL group (n=40)***	***High PAL group (n=40)***
Age (yr)	21.05 ± 1.65	20.98 ± 1.44	21.12 ± 1.67
Height (cm)	161.28 ± 4.46	163.10 ± 4.81	162.29 ± 4.88
Weight (kg)	59.17 ± 10.05	63.19 ± 15.84	58.73 ± 7.63
Body mass index (kg/m^2^)	22.68 ± 3.24	23.68 ± 5.48	22.31 ± 2.87
Total physical activity (MET-minutes/week)	282.99 ± 206.17	1167.15 ± 361.33	3467.85 ± 1852.11

Data are presented as means ± standard deviations

PAL, physical activity level; MET, metabolic equivalent of task

**Table 2:** The differences in the 3 physical activity level groups according to skin health outcomes

***Group***	***Low PAL (n=40)***	***Middle PAL (n=40)***	***High PAL (n=40)***	***Overall* F**	***Overall* p**
Pores (%)	48.43±9.12	46.98±9.70	46.48±8.88	0.481	0.619 N/S
Wrinkles (%)	23.23±16.05	17.83±11.78	13.90±7.68 ^##^	5.777	0.004 **
Pigmentation with ultraviolet light (%)	11.68±6.96	10.90±6.43	9.18±4.36	1.806	0.169 N/S
Pigmentation with polarizing light (%)	10.65±7.00	11.25±7.88	9.23±5.75	0.901	0.409 N/S
Sebum level (total count)	57.58±62.25	110.58±186.06	71.78±97.32	1.883	0.157 N/S
Porphyrin content (%)	25.93±18.58	32.88±24.87	26.80±18.51	1.316	0.272 N/S
Skin tone (%)	56.95±2.29	57.13±3.74	56.45±2.43	0.587	0.558 N/S

Data are presented as means ± standard deviations

PAL, physical activity level

N/S, not significant,

** *P*<0.01; tested by one-way analysis of variance,

## *P*<0.01; compared with low PAL group; Tukey's post-hoc testing

We concluded that even though the high PAL group showed a positive outcome with respect to wrinkles, overall, the PAL has little or no effect on skin health outcomes in Korean female college students. From this result, PAL could not be considered a preventive factor in skin health. Nevertheless, as PAL and exercise are different and independent concepts, future studies are required to examine the effect of regular exercise, but not PAL, on skin health.
